# Supplementary material for: A Comprehensive Assessment of Ultraviolet-Radiation-Induced Mutations in Flammulina filiformis Using Whole-Genome Resequencing
Source: J Fungi (Basel). 2024 Mar 20;10(3):228. doi: 10.3390/jof10030228 (PMC10971301; doi:10.3390/jof10030228)
Supplement: Supplementary file 1 [file jof-10-00228-s001.zip › Supplementary Material S8/KEGG annotation/out/64381550635650.os/KO/out_map/map03430.html]

KEGG PATHWAY: Mismatch repair - Reference pathway


|  |  |
| --- | --- |
| **Mismatch repair - Reference pathway** |  |

[
Pathway menu
| Organism menu
| Pathway entry
| Show description
| User data mapping
]

DNA mismatch repair (MMR) is a highly conserved biological pathway that plays a key role in maintaining genomic stability. MMR corrects DNA mismatches generated during DNA replication, thereby preventing mutations from becoming permanent in dividing cells. MMR also suppresses homologous recombination and was recently shown to play a role in DNA damage signaling. Defects in MMR are associated with genome-wide instability, predisposition to certain types of cancer including HNPCC, resistance to certain chemotherapeutic agents, and abnormalities in meiosis and sterility in mammalian systems.
The Escherichia coli MMR pathway has been extensively studied and is well characterized. In E. coli, the mismatch-activated MutS-MutL-ATP complex licenses MutH to incise the nearest unmethylated GATC sequence. UvrD and an exonuclease generate a gap. This gap is filled by pol III and DNA ligase. The GATC sites are then methylated by Dam. Several human MMR proteins have been identified based on their homology to E. coli MMR proteins. These include human homologs of MutS and MutL. Although E. coli MutS and MutL proteins are homodimers, human MutS and MutL homologs are heterodimers. The role of hemimethylated dGATC sites as a signal for strand discrimination is not conserved from E. coli to human. Human MMR is presumed to be nick-directed in vivo, and is thought to discriminate daughter and template strands using a strand-specific nick.

|  |  |
| --- | --- |
| Reference pathway | 100% |
